# Intelligent systems for additive manufacturing-based repair in remanufacturing: a systematic review of its potential

**DOI:** 10.7717/peerj-cs.808

**Published:** 2021-12-10

**Authors:** Siti Syahara Mad Yusoh, Dzuraidah Abd Wahab, Hiyam Adil Habeeb, Abdul Hadi Azman

**Affiliations:** 1 Department of Mechanical and Manufacturing Engineering, Faculty of Engineering and Built Environment, Universiti Kebangsaan Malaysia, Bangi, Selangor, Malaysia; 2 Centre for Automotive Research, Faculty of Engineering and Built Environment, Universiti Kebangsaan Malaysia, UKM Bangi, Selangor, Malaysia; 3 Technical College Al-Mussaib, Al-Furat Al-Awsat Technical University, Babylon, Iraq

**Keywords:** Additive manufacturing, Artificial intelligence, Repair and restoration, Conventional methods, Intelligent systems

## Abstract

The conventional component repair in remanufacturing involves human decision making that is influenced by several factors such as conditions of incoming cores, modes of failure, severity of damage, features and geometric complexities of cores and types of reparation required. Repair can be enhanced through automation using additive manufacturing (AM) technology. Advancements in AM have led to the development of directed energy deposition and laser cladding technology for repair of damaged parts and components. The objective of this systematic literature review is to ascertain how intelligent systems can be integrated into AM-based repair, through artificial intelligence (AI) approaches capable of supporting the nature and process of decision making during repair. The integration of intelligent systems in AM repair is expected to enhance resource utilization and repair efficiency during remanufacturing. Based on a systematic literature review of articles published during 2005–2021, the study analyses the activities of conventional repair in remanufacturing, trends in the applications of AM for repair using the current state-of-the-art technology and how AI has been deployed to facilitate repair. The study concludes with suggestions on research areas and opportunities that will further enhance the automation of component repair during remanufacturing using intelligent AM systems.

## Introduction

### Remanufacturing as a strategy for product life cycle extension

The circular economy (CE) offers an alternative perspective for recovery of durable technical systems and returning them to productivity as part of a sustainable future. It advocates designing out waste and pollution, retaining products and materials in use, and regenerating natural systems (Kirchherr, Reike & Hekkert, 2017). Gan & Pujawan (2014) noted that rapid developments and innovations in science and technology have shortened the life cycle of products. This phenomenon is not in line with the principles of CE that promote sustainability through product life cycle extension. Environmental sustainability will be adversely affected by the overconsumption of natural resources and energy, causing a rapid increase in landfill waste. The principles are set to replace the ‘take–make–dispose’ concept of a linear economy, which relies heavily on resources and energy. The emphasis on regeneration and restoration of durable products to extend their useful life has become a subject of interest in product development, as it describes the recovery and restoration of products through strategies such as reuse, repair, remanufacturing or recycling (Macarthur, 2015).

Hollander & Bakker (2012) outlined five modes for product life cycle extension: repair, recycle, maintenance, upgrade and remanufacturing. Based on a study on waste management for sustainability, Fedotkina, Gorbashko & Vatolkina (2019) reiterated the importance of reuse, remanufacturing and recycling as enablers of CE. Awareness of the principles of CE will help create ‘closing the loop’ in product life through repair and restoration (Delos Rios & Charnley, 2017). Researchers that have compared remanufacturing with other end-of-life (EoL) recovery strategies such as recycling, suggested that remanufacturing is less energy intensive and more profitable (Taleizadeh, Alizadeh-Basban & Akhavan, 2019; Tam, Soulliere & Sawyer-Beaulieu, 2019; Kalverkamp & Raabe, 2018; Wakiru et al., 2018).

To date, remanufacturing is the most value-added, resource-efficient strategy for EoL product recovery compared with recycling or reuse, as it enables products to be given a new life (Hatcher, Ijomah & Windmill, 2013). Remanufacturing has been defined as the process of returning a used product to a ‘like new’ condition in terms of its original specifications and given a matching warranty (Ijomah, 2010). Remanufacturing involves a series of activities, namely, disassembly, cleaning, inspection, repair, reassembly and testing on an EoL product before it can be reused in a new life cycle (Matsumoto et al., 2016). Recent studies indicated that remanufacturing is a good alternative towards enabling CE; this will in turn, sustain the economy, the society and the environment (Andrews, 2015; Parker et al., 2015). Based on the study by Van Loon & Van Wassenhove (2017) on manufacturing and remanufacturing of a chassis for automotive industry, the CO2 emission for a new chassis is 11.23 kg per item, whereas it is only 7.5 kg for remanufacturing. An earlier study by Biswas & Rosano (2011) showed that the emission of greenhouse gas for the production of a new compressor is approximately 1,590 kg CO2, whereas the emission for compressor remanufacturing is approximately 110–168 kg CO2. The ratio of energy benefits between remanufacturing and manufacturing is in the range of 2%–25% for engine diesel remanufacturing. An eco-efficiency analysis of remanufacturing and manufacturing of a cylinder block has shown that a remanufactured cylinder block shows better performance, which is 62% compared with a newly manufactured component in addition to reduction in price, use of material and air emissions (Afrinaldi et al., 2017).

Remanufacturing maintains a product’s original functions and applications, with slight differences in its form, configuration or construction to support functions equivalent to those of the virgin product (Soh, Ong & Nee, 2014). Through remanufacturing, the value of a used product can be retained (Aziz et al., 2016). Extending a product’s life cycle not only reduces the product’s effect on the environment but also increases its economic value (Hollander & Bakker, 2012). According to Amelia et al. (2009), resources that have undergone the recovery phase enter a new life cycle, thus reducing or eliminating the usage of virgin materials. This finding was also explored in prior studies by Gray & Charter (2006), who stated that remanufacturing reduces energy and natural material consumption and costs. Kara, Pornprasitpol & Kaebernick (2006) noted that remanufacturing reduces the environmental influence of a product and improves eco-efficiency. For a successful remanufacturing, a product should possess a stable technology for more than one cycle ahead (Amelia et al., 2009). Yusop, Wahab & Saibani (2015) described remanufacturing as one of the factors in achieving environmental sustainability and has a direct positive effect on economic growth in terms of cost saving and profit obligations. Aprilia et al. (2018) noted that high-value components such as turbine blades, impeller and shafts are preferred to be remanufactured rather than replaced with new ones.

This study focuses on the repair and restoration process of remanufacturing. Despite the significance of remanufacturing as one of the recovery strategies in CE, remanufacturing is labour intensive because it is highly dependent on skilled and experienced workers and the use of conventional tools for component repair and restoration. Therefore, automating the process is crucial such that productivity and process consistency can be controlled and improved. Aprilia et al. (2018) noted that manual remanufacturing is prone to human error, inconsistency in output quality and labour intensive with a high cost. Automating the activity is crucial to enhance productivity and process consistency.

Literatures on the use of advanced additive manufacturing (AM) technology such as direct energy deposition and cold spray in component repair have grown in recent years. However, these technologies have yet to be integrated with the capability to replicate human judgement and decision-making performed during conventional remanufacturing repair and restoration in order to provide intelligence in machine learning (ML).

The conventional repair and restoration in remanufacturing demands skilled and experienced workers who can make decisions on whether incoming products are worthy of remanufacturing. For example, during core inspections, workers have to assess the conditions of the cores that include types of damage, severity of damage, geometric complexity and availability of the right tools. Each incoming core has different levels of conditions and geometrical complexity, causing vagueness and uncertainties in decision making. Human decision making for repair and restoration is influenced by factors such as product features and geometry, mode and severity of damage and failure as well as process and system capabilities. An intelligent system based on artificial intelligence (AI) can capture these information and knowledge on design and process that facilitates knowledge sharing and ML for automation. Models for decision making, prediction and optimisation can be developed through AI techniques, resulting in a more consistent, efficient decision making. Representing knowledge and heuristics on repair and restoration through hybridised techniques of fuzzy logic, case-based reasoning (CBR), neural networks will enable various improvements in quality and reliability of products and process efficiency.

This study provides a systematic review on the potential of AM in automating remanufacturing repair through an integration with AI-based intelligent systems. The first part of the findings reports on remanufacturing as one the strategies of product life cycle extension in CE, followed by an overview of conventional methods and automated process for repairing components. For the purpose of this review, the automated process is focused on the application of AM technology. The review also provides a coverage of literature that address the applications of AI in component repair. The review concludes with research gaps in the field of study and suggestions for further research and exploration for the integration of AI-based intelligent systems in AM for component repair.

### Remanufacturing repair and restoration: practices and challenges

**Figure 1:**
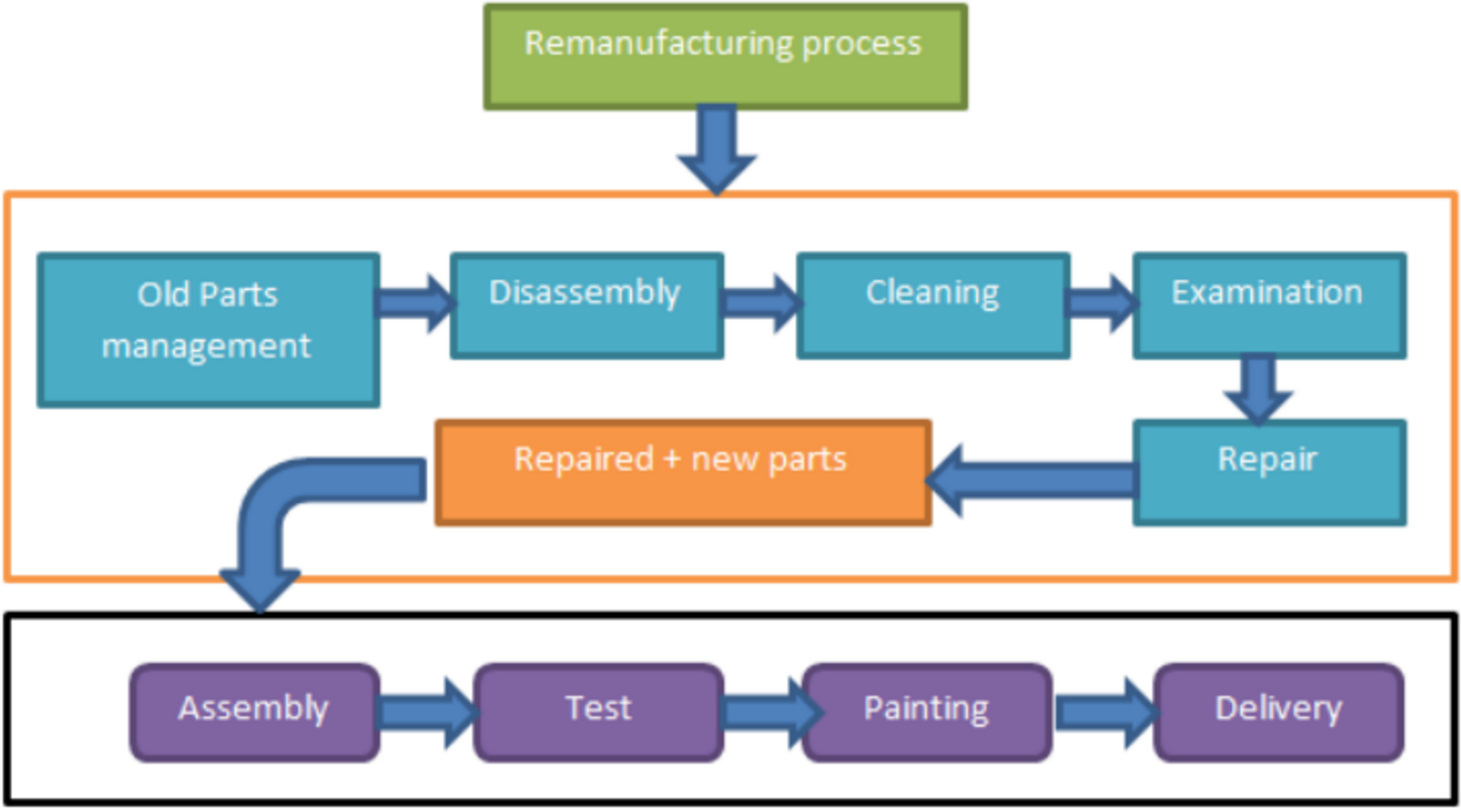
Remanufacturing process (Jiang, 2016). The general processes involved in remanufacturing.

Remanufacturing involves several processes: sorting, disassembly, inspection, cleaning, repair and restoration, reassembly and testing. These processes are commonly performed using conventional methods by highly skilled, experienced workers (Hatcher, Ijomah & Windmill, 2013; Yilmaz, Gindy & Gao, 2009; Wahab et al., 2018). Figure 1 depicts the general processes involved in remanufacturing.

Despite the importance of remanufacturing in terms of socio–economic gains, companies are facing challenges in implementing efficient remanufacturing because activities such as disassembly, cleaning and inspection of damaged components and repair rely on conventional methods.

Generally, repair and restoration are aimed at returning EoL components to their original form before entering the subsequent life cycle. Kin, Ong & Nee (2014) noted that manual restoration consists of a sequence of processes to ensure that cores are free from damages. Component repair may involve the removal of surface and shape defects using conventional processes such as turning, milling, drilling and grinding, after which a material or a surface is replaced using other processes such as welding and powder coating. Next, material properties are restored through conditioning, such as heat treatment or case hardening involving carburising. Finally, parts undergo surface finishing to achieve dimensional tolerances using processes such as grinding, hard turning and burnishing (Kin, Ong & Nee, 2014). According to Andrew-Munot & Ibrahim (2014), manual repair and restoration of damaged parts with complex designs require multiple repair processes, such as cutting, welding and trimming. Priyono, Ijomah & Bititci (2015) stated that repair and restoration may involve treatment to the surface, such as blasting and building up worn-out parts by means of metal spraying and welding.

Metal deposition on damaged surfaces is normally carried out using arc welding, gas tungsten welding and other methods (Wilson et al., 2014). According to Widener, Ozdemir & Carter (2018) and Subramoniam, Huisingh & Chinnam (2009), these workers have to decide whether a product should be repaired or replaced during remanufacturing. Making the right decisions is crucial when selecting the appropriate repair and restoration because they depend on the type of damage of each component (Lahrour & Brissaud, 2018), whether products should be remanufactured (Paterson, Ijomah & Windmill, 2017), selection of toolings and techniques in the disassembly (Soh, Ong & Nee, 2014) and analysing variables such as successful remanufacturing rate, number of product life cycles and environmental performance (Yang et al., 2015).

Casper & Sundin (2018) noted the difficulties in repairing cores with a complex design because it requires highly skilled, experienced workers. Issues such as lack of consideration on design for remanufacturing during the early design phase of products have led to remanufacturing inefficiency (Kurilova-Palisaitiene & Sundin, 2014). Matsumoto et al. (2016) pointed out several challenges in remanufacturing automotive parts and photocopiers such as product and process design to facilitate remanufacturing, engineering and optimisation. Process design such as the method for joining parts is crucial in achieving efficient remanufacturing. Yang et al. (2015) highlighted problems in remanufacturing, such as high degree of damage to incoming EoL components, lack of technology and equipment, and substantial involvement of manual labour. The processes of disassembly, cleaning and inspection of core parts also face difficulties due to the presence of highly contaminated oil, oxide and operational liquid (Yang et al., 2015).

Shahbazi, Johansen & Sundin (2021) have reported on several advantages of using automation in remanufacturing. According to the authors, separating the material of the component into different sections is easier using an automated system that increases efficiency and productivity, and reduces lead time as well as the cost involved.

## Survey methodology

This review is part of a research funded by the Ministry of Higher Education, Malaysia (JPT(BKPI)1000/016/018/25(72)) aimed at integrating product design and process efficiency during AM repair and restoration in remanufacturing through the applications of AI based systems. The review methodology is an adaptation of the methods used by Linares-Espinós et al. (2018) and Campbell et al. (2020). This work starts by identifying research questions that will answer the primary objectives and address the scope of this review. Linares-Espinós et al. (2018) stated that a review should be carried out when developing and planning to reduce biased, irrelevant and low-quality studies. The findings from the review are analysed and synthesised accordingly. An exploratory search on the related topic is carried out using relevant publications to fulfil the requirements of the scope of the review. Once the research questions are developed, the related hypotheses are formulated as follows: ‘If AM technology is applied to the current remanufacturing activities, then difficulties in the manual process can be solved’, and ‘If an intelligent system is integrated with AM technology, the repair process can be performed with minimal human intervention’. The formulated hypotheses have led to a more focused, systematic search and analysis of the research topic.

Challenges and difficulties in conventional repair during remanufacturing are highlighted in this paper. The applications of AM technology as the way forward in component repair during remanufacturing are thoroughly searched and discussed in the context of a sustainable technology in supporting CE. In the last part, the applications of intelligent systems based on AI in component repair are reviewed and discussed as the way forward in supporting the remanufacturing industry. Intelligent systems in this context include the applications of AI techniques in recognising damaged areas on parts, predicting part distortion, detecting part failure and decision making during repair.

Okoli & Schabram (2010) noted that several steps should be followed when conducting a systematic literature review. The first step is to select research questions, bibliographic or article databases, websites or other sources, and select the right search terms. This step is followed by applying practical screening criteria for the relevant literature and the methodological screening criteria. The final step is to analyse and synthesise the findings from the literature review. Table 1 shows the research questions from which the search terms were identified and used to obtain the related publications on ‘remanufacturing’, which was firstly focused on the manual process, followed by automation using AM to search information on the current technology that is generally in use. The term ‘repair and restoration using additive manufacturing’ was then used to narrow the scope of the search. This study emphasises the application of AI technology within AM repair in remanufacturing systems and its implementation. Therefore, the terms ‘artificial intelligence’ and ‘machine learning’ were used to search the related information. Remanufacturing is highly related to sustainability and CE together with ‘life cycle’, and the following search terms were used: ‘sustainability’, ‘circular economy’, ‘life cycle’, ‘strategy for life cycle extension’, ‘intelligent manufacturing’, ‘intelligent systems in remanufacturing’, ‘intelligent systems in reparation’, ‘AI method for reparation’, ‘AI application in reparation’, ‘additive manufacturing in circular economy’ and ‘artificial intelligence in circular economy’.

**Table 1:** The research questions from which the search terms were identified and used to obtain the related publications.

Issues in remanufacturing	Research questions
Difficulties or challenges in manual repair and restoration of components during remanufacturing.	Question 1: What are the activities of repair and restoration that can be automated using enabling technologies?
The possibility of deploying AM technology for repair and restoration process to overcome limitations in conventional methods.	Question 2: How can AM be deployed in repair and restoration to enhance remanufacturing efficiency?
The process of repair and restoration of EoL components is highly dependent on human judgement and decision-making.	Question 3: How can AM-based repair and restoration be made more intelligent through AI and machine learning?

### Research questions

**Figure 2:**
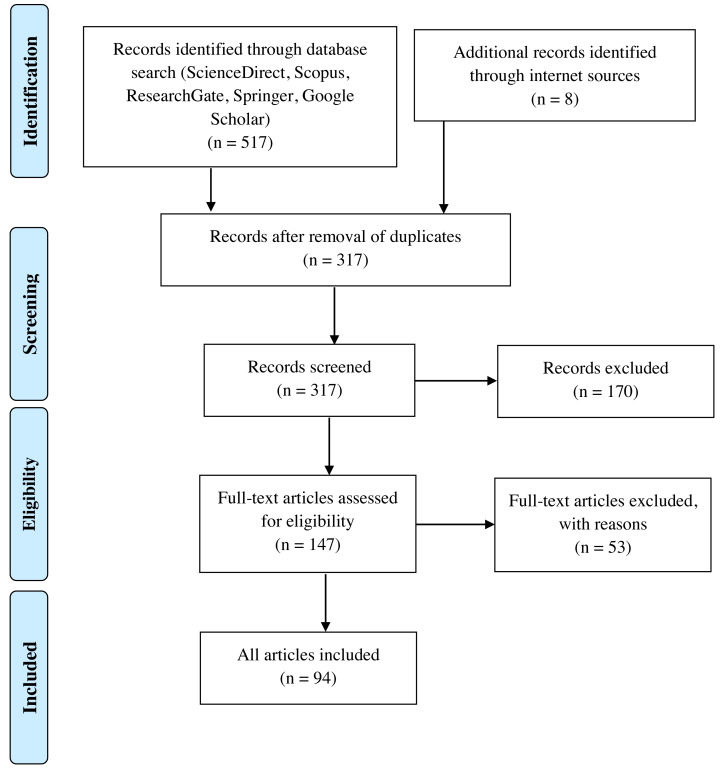
Process flow for a systematic review of related articles. Illustration of the process flow for the systematic review of related articles.

To obtain the full text of the publications from those search terms, databases of several publishers, such as Elsevier (http://www.sciencedirect.com and http://www.scopus.com), Research Gate (http://www.researchgate.net) and Springer (http://www.springer.com) were used. Google Scholar was also used to search for journal publications and conference proceedings. For the purpose of the systematic review, articles were selected from 2005 to 2021 to ensure that the review is up to date, and research trends can be determined throughout the specified timeline. Once the related publications were obtained based on the search terms, a two-level screening process was carried out starting with the first level screening which focused on the title and abstract. The second level screening was carried out based on a full-text review. After an initial screening of 517 citations and removal of duplicates based on title and abstract, 317 citations were obtained. The items were further screened for eligibility based on a full-text review resulting in 147 full-text articles; the 170 other articles were excluded because they were out of relatable context. Articles excluded will be counted to complete the Preferred Reporting Items for Systematic Reviews and Meta-Analyses (PRISMA) flow diagram as shown in Fig. 2. The articles were screened further to meet the inclusion criteria which is AM and AI technology applied in any country, any AM and AI method applied in enhancing the remanufacturing activity, automated repair and restoration using enabling technologies and intelligent repair and restoration through AI and ML. This will include the studies of experiences, perceptions, opinions on potential use and related case studies, resulting in 94 items.

Only articles published in English were used as reference to avoid language bias (Suhariyanto, Wahab & Rahman, 2017). The review was focused more on case studies in industries such as aerospace, automotive, mould and die, and archaeology, in which the application of AM has been reported. In addition, AM technologies other than metal AM were excluded from this review. Figure 2 illustrates the process flow for the systematic review of related articles.

## Results

### Repair and restoration in remanufacturing

Component repair during remanufacturing can be broadly classified into conventional and automated processes. The process begins after cores are disassembled, sorted and inspected. Kin, Ong & Nee (2014) noted that returned products might exhibit defects ranging from minor scratches to considerable damage; thus, inspection and sorting are required to filter valuable cores. Common types of defects are cracks, scratches, nicks and burrs as well as burnt or corroded regions. The following sections discuss remanufacturing repair and restoration that are classified as conventional methods and automated methods using AM technology.

### Conventional repair and restoration

Repair and restoration in remanufacturing is aimed at returning parts or components to their original forms or dimensions. Researchers such as Kin, Ong & Nee (2014) have discussed methods for the removal of surface and shape defects prior to material addition using processes such as turning, milling, drilling and grinding. Welding, one of the most common conventional processes, was reported by Kim et al. (2012) as the method for repairing cracks on the surface of an engine block head. Bennett et al. (2019) noted that welding has also been used extensively in the repair of aircraft components, such as gas turbine blades. The setbacks of welding have also been reported by researchers in the field of study. Amongst the setbacks are exposure of the parent metal to high temperatures causing the formation of heat-affected zone that alters the properties of materials (Ramkumar et al., 2014) and the need to remove material before the damaged area can be restored (Balakrishnan & Seidlitz, 2018; Walachowicz et al., 2017). Kurilova-Palisaitiene, Sundin & Poksinska (2018) noted that compared with manufacturing, remanufacturing involves a higher proportion of manual work that may lead to a prolonged lead time, rendering inefficiency in remanufacturing. This labour-intensive process that directly imparts factors related to human limitations affect the flow of the repair process; thus, proper restoration measures are needed (Balakrishnan & Seidlitz, 2018).

### Automating repair using additive manufacturing

Today, advancement in AM technology has expanded its role from part manufacturing to part repair and restoration. Sauerwein et al. (2020) stated that AM supports circular design strategies by creating opportunities in extending a product lifespan, for example, enabling the reparation and upgrading of products even though the product is not designed for ease of repair or upgrade. Moreover, broken parts of products can be reproduced by digital production to support reparation. According to Huang et al. (2014), AM allows the creation of complex geometries that will lead to reduced energy consumption and material usage, and increased product functionality and simplified assembly lines.

Metal AM has two types of processes: direct and indirect. The direct process is laser beam melting, also known as selective laser melting (SLM), laser cladding and laser-engineered net shaping (LENS) (Boschetto et al., 2019). Directed metal deposition (DMD), also known as directed energy deposition (DED), consists of several methods, such as LENS, laser cladding and cold spray (Herzog et al., 2016). The indirect process refers to 3D printing, which includes selective laser sintering and stereolitography (Boschetto et al., 2019).

Yeo, Pepin & Yang (2016) noted that to reduce the challenges and enhance remanufacturing, automated systems should be deployed for critical repair and restoration processes. Several authors have highlighted the benefits of AM in terms of efficiency, time saving and its ability to work with intricate designs compared with conventional techniques (Liu et al., 2017; Ko, Moon & Hwang, 2015). AM, as one of the enabling technologies of Industrial Revolution (IR) 4.0, could provide potential benefits in terms of sustainability in remanufacturing because it can extend a product’s life cycle (Den Boer, Lambrechts & Krikke, 2020; Ford & Despeisse, 2016).

**Figure 3:**
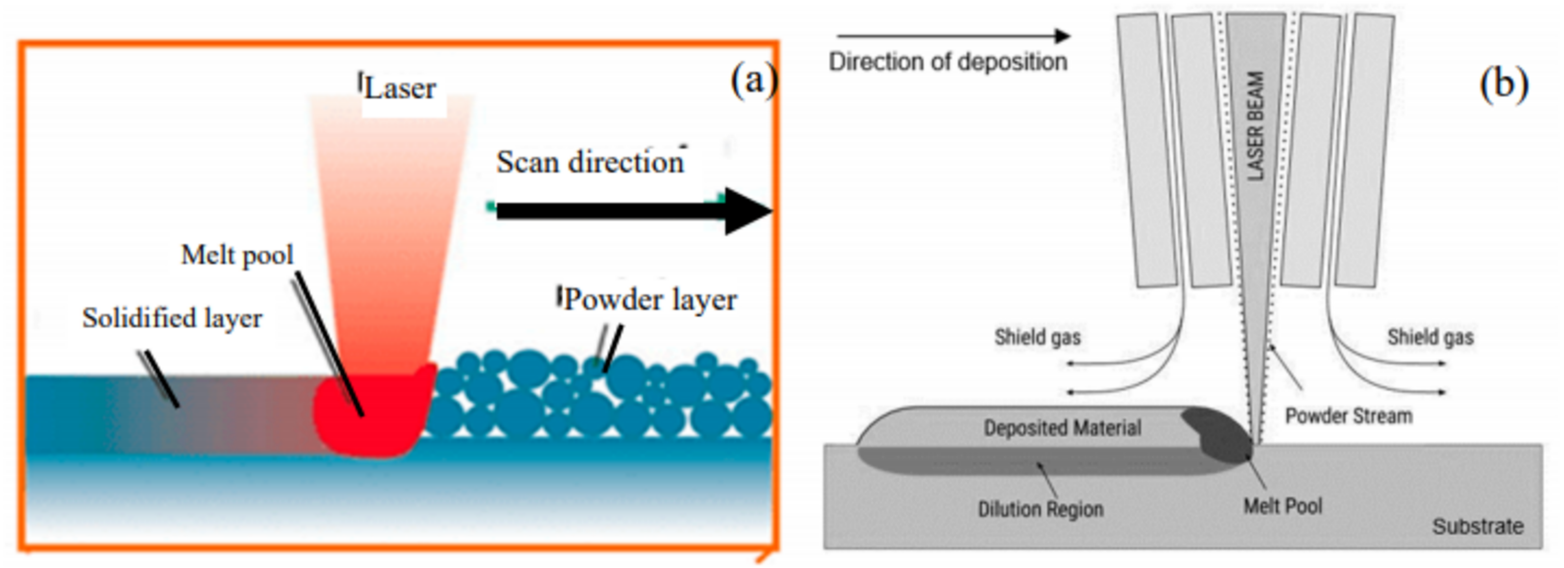
Schematic diagram of (A) PBF and (B) powder-based directed deposition (Saboori et al., 2017). Schematic diagram of PBF and DED.

To date, AM technologies such as powder bed fusion (PBF) and DED have paved the way for AM applications in repair and restoration. The technology has also been recognised as environmentally sustainable due to its ability to minimise energy and material consumption. Figure 3 shows a schematic diagram of PBF and DED.

**Figure 4:**
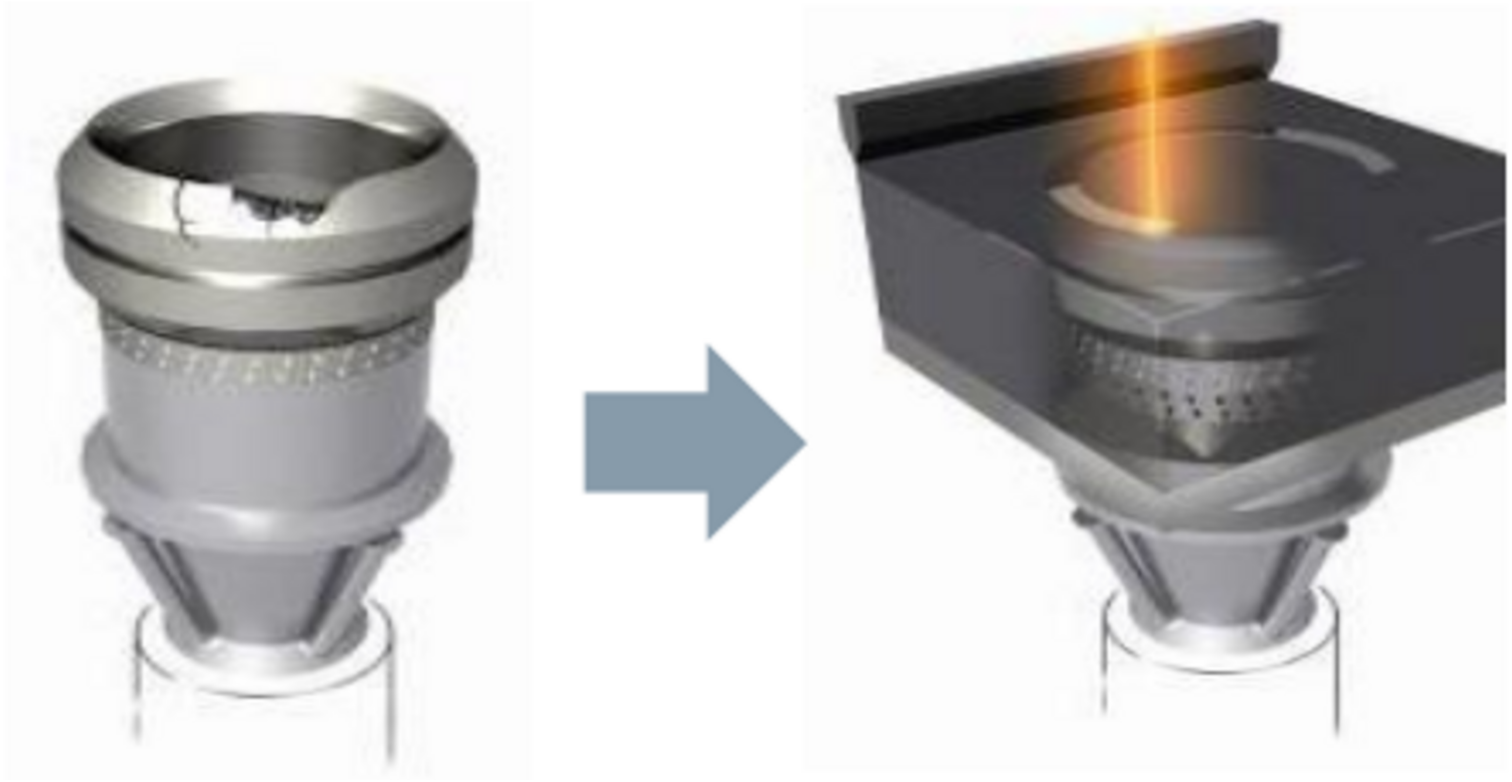
Repair of a gas turbine burner tip. Example of a component that can be repaired using PBF is the burner tip of a gas turbine using SLM technology.

PBF has been widely used in manufacturing replacement components on demand as well as reparation of damaged components. Generally, PBF is a process of layer-by-layer building of material by channelling laser beams into a thin layer of powder deposited on a fusion bed (Okaro et al., 2019). However, its limitation is that new materials need to be in the form of a planar surface within the tolerance of the powder layers (Matsumoto et al., 2016). An example of a component that can be repaired using PBF is the burner tip of a gas turbine using SLM technology, as shown in Fig. 4 (Navrotsky, 2019). The tip of the burner is machined to remove the damaged area before it is placed into the powder bed.

Matsumoto et al. (2016) reported on the use of DED for component repair in remanufacturing. The process uses a focused thermal energy beam to melt and fuse materials. Material in powder form is added by injecting a flow of powder and inert gas into the fusing zone through a nozzle or the mechanical feeding of wire from a spool. Depositing the material onto the damaged surface is easier compared with the PBF technology. It can also add more wear-resistant material to the outer surface than the original material, thus improving the functionality of the remanufactured part. The American Society for Testing and Materials classified AM technology into seven categories, namely, material extrusion, material jetting, binder jetting, DED, PBF, sheet lamination and VAT polymerisation (Huang et al., 2016). This literature review focuses on AM technologies that have been applied in component repair and restoration, namely, PBF and DED. Leino, Pekkarinen & Soukka (2016) noted that DED is expected to be one of the enabling technologies in CE that can support repair, refurbishing and remanufacturing activities. A comparison between DED and PBF in terms of technology and repair principles is presented in Table 2.

**Table 2:** Comparison between DED and PBF in terms of technology and repair principles.

	Powder bed fusion (PBF)	Directed energy deposition (DED)	Author
**Definition**	A layer-by-layer building of material by channelling laser beams onto a thin layer of powder deposited on a fusion bed.	Using a focused thermal energy beam to melt and fuse materials. The material is added by injecting a flow of powder and inert gas into the fusing zone through a nozzle or the mechanical feeding of wire from a spool.	Matsumoto et al. (2016) Widener et al. (2016)
**Types of process**	Selective laser melting (SLM), direct metal laser sintering (DMLS), selective laser sintering (SLS) and electron beam melting (EBM).	Laser engineered net shaping (LENS), shaped metal deposition (SMD), laser cladding and cold spray.	Kok et al. (2017)
**Repair principles**	The damaged part is scanned to obtain the point cloud and converted to a 3D CAD model. The slice is then generated from the new solid, and the damaged parts are remanufactured by building new parts.	The restoration process involves four stages: pre-machining of the damaged zone, material deposition, post-machining on back-filling material and performance testing.	Liu et al. (2016); Lyalyakin, Kostukov & Denisov (2016)
**Example of application in repair and restoration**	Corroded transmission gearboxes in aircrafts, corroded air pump housings in the automotive industry, valve actuators in the marine industry and moulds.	Gas turbine burner tip, sewing machine gears.	Matsumoto et al. (2016); Widener et al. (2016); Kok et al. (2017); Lyalyakin, Kostukov & Denisov (2016); Yin et al. (2018); Lee et al. (2007); Okaro et al. (2019); Greving et al. (2016); Wuest et al. (2016).

### Comparison between DED and PBF technology

Based on extensive literature, a number of articles that addressed the application of AM in repair and restoration were identified. Table 3 provides a summary of selected articles that discussed the applications of AM for various types of reparation.

**Table 3:** Summary of articles on the applications of AM in repair and restoration. Summary of selected articles that discussed the applications of AM for various types of reparation.

No	Author (s)	Topic	Year published	Description of the published work
1	Lee et al.	Repair of Damaged Mold Surface by Cold-Spray Method	2007	• Reparation process of a mould using cold spray, focusing on the properties of the mould material (aluminium). • Comparison of the wear properties of the original mould and repaired mould using a ball-on-disk test. • Evaluation of parts from undamaged mould and repaired mould made from injection moulding process
2	Wilson et al.	Remanufacturing of Turbine Blades by Laser Direct Deposition with Its Energy and Environmental Impact Analysis	2014	•Restoration of turbine blades for aerospace applications using laser direct deposition (LDD), focusing on geometry. • The environmental impact of LDD was analysed using a life cycle analysis (LCA) software. • Tensile testing of restored blade parts was reported.
3	Kamrani	Direct Laser Deposition for Re-Manufacturing of Components	2014	• The capability of AM in manufacturing and remanufacturing of worn-out components. • The process of restoration using direct laser deposition (DLD) and the post-processing involved.
4	Buican, Oancea & Manolescu	Remanufacturing of Damaged Parts Using Selective Laser Melting Technology	2014	• Reparation of damaged gears of a sewing machine using selective laser melting (SLM) technology.
5	Lyalyakin Kostukov & Denisov	Special Features of Reconditioning the Housing of a Caterpillar Diesel Oil Pump by Gas-Dynamic Spraying	2016	• Reconditioning process of oil pump housing using cold gas dynamic spraying of powder materials. • Post-processing of the repaired component.
6	Widener et al.	Application of High-Pressure Cold Spray for an Internal Bore Repair of a Navy Valve Actuator	2016	• Reparation of a navy valve actuator using high-pressure cold spray. • Challenges in applying cold spray for the reparation of components. • Performance requirements for the repaired component, such as porosity, tensile testing and bonding testing.
7	Liu et al.	Environmental Benefits of Remanufacturing: A Case Study of Cylinder Heads Remanufactured	2016	• Environmental impacts of the reparation of cast iron cylinder head blocks using laser cladding technology. • Comparison of environmental impact between remanufactured and new cylinder head block manufacturing in terms of resources and energy consumptions. • The process of remanufacturing a cracked cylinder head and analysis of the environmental impact using LCA.
8	Walachowicz et al.	Comparative Energy, Resource and Recycling Lifecycle Analysis of the Industrial Repair Process of Gas Turbine Burners Using Conventional Machining and Additive Manufacturing	2017	• Comparison between reparation of gas turbine burners (Siemens) using conventional machining and AM in terms of environmental impact. • The process of repairing burners using laser beam melting (LBM). • Life cycle analysis of the repaired burners using LBM.
9	Yin et al.	Cold Spray Additive Manufacturing and Repair: Fundamentals and Applications	2018	• Working principles and parameters for cold spray technology, such as propulsive gas, powder feeder, nozzles and spray angle. • Post processing for cold spray technology which is conventional machining. • Restoration method using cold spray technology for a variety of damages, such as corrosion and erosion, mechanical damage and metal sheet damage. • Challenges in applying cold spray technology in a variety of applications.

### Summary of articles on the applications of AM in repair and restoration

**Figure 5:**
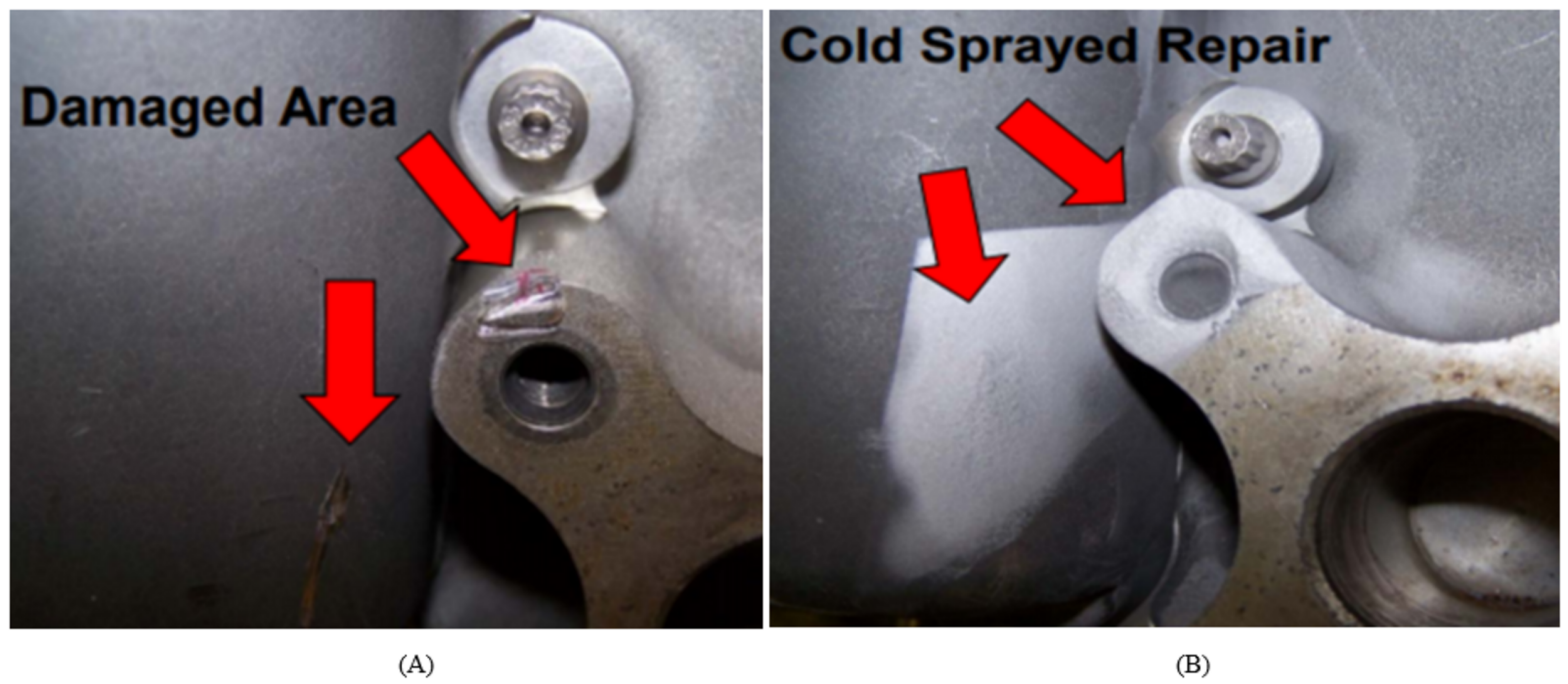
Application of cold spray technology (A) Before repair (B) After repair. The cold spray technology that has been used for repairing damaged and corroded materials.

Yin et al. (2018) investigated the use of cold spray technology for repair and restoration of damaged components. Originally developed as a coating technology in the 1980s, cold spray technology is a solid-state material deposition using high-temperature compressed gas, typically nitrogen, air or helium, as a propulsive gas to accelerate a powder feedstock to a high velocity and induce deposition when the powder impacts onto a substrate. Cold spray has a high potential for repairing damaged components due its ability to avoid thermal damage to the material. The technology has been used in various fields for repairing damaged and corroded materials, as shown in Fig. 5. Examples of components that have been repaired using cold spray are corroded transmission gearboxes in aircrafts, corroded air pump housings in the automotive industry, valve actuators in the marine industry and damaged moulds (Widener et al., 2016; Kok et al., 2017; Lyalyakin, Kostukov & Denisov, 2016; Yin et al., 2018; Lee et al., 2007; Okaro et al., 2019).

**Figure 6:**
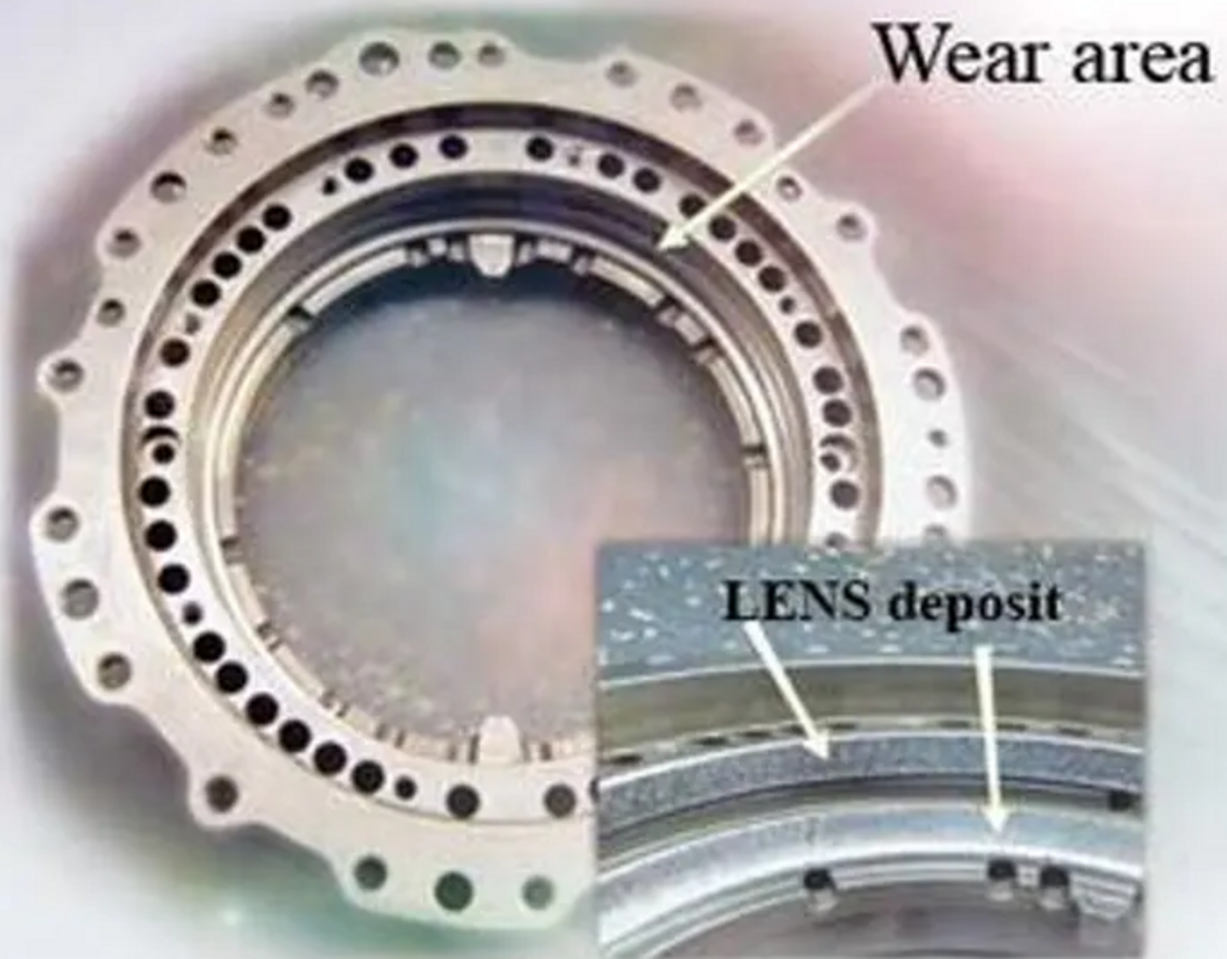
Bearing housing repair using LENS technology. Examples of damaged components that have been repaired using LENS.

**Figure 7:**
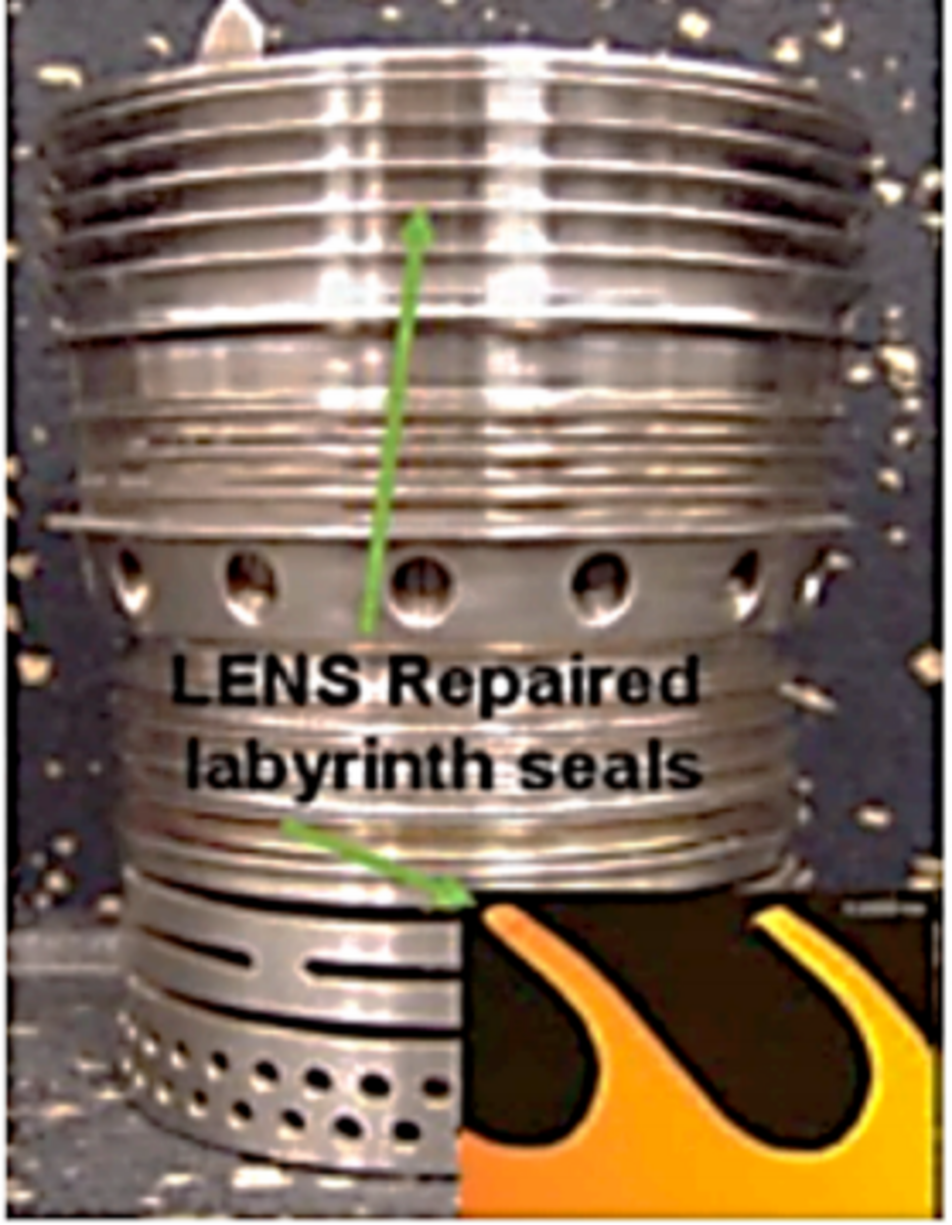
Compressor seal repair using LENS technology. Examples of damaged components that have been repaired using LENS.

LENS is characterised as a disruptive additive process that can be employed in a variety of processes for repair and free-form fabrication. This technology can be used for repairing worn-out components, building near net-shaped free forms directly from CAD files and cladding materials. LENS works by melting the surface of the target using a focused laser beam to generate a small molten pool of the base material. Examples of damaged components that have been repaired using LENS are bearing housings, drive shafts, gas turbines, compressors and drive couplers (Mudge & Wald, 2007). Figures. 6 and 7 show a bearing housing and a compressor seal repaired using LENS.

Kamrani (2014) studied component repair during remanufacturing using direct laser deposition technology, known as LENS, and laser cladding. Typical damages of remanufacturable components include abrasion, seizure, fretting, erosion, cavitation and fracture spalling. According to Kamrani (2014), the most severe type of damage is abrasive defect caused by small particles penetrating and indenting the surface of a material. AM remanufacturing is preferred to traditional machining, as less time is required to work on complex models or geometries, and no waste material is created. LENS and laser cladding can be applied in remanufacturing for various industries such as automotive, aerospace, medical, and oil and gas (Kamrani, 2014; Linton, Klassen & Jayaraman, 2007). Kamrani (2014) studied the potential of direct laser deposition in component repair based on damage types; however, the processes involved in repairing these components were not addressed. Linton, Klassen & Jayaraman (2007) discussed the role of AM in product life extension that is now the way forward in IR 4.0.

Liu et al. (2016) discussed the use of laser cladding during remanufacturing of damaged cylinder heads made of cast iron. The damage occurs as a crack between the valve seats of the air inlet and the exhaust port on top of the cylinder head. Liu et al. (2016) also made a comparison between the use of conventional welding and laser cladding for reparation, which resulted in laser cladding being the more favourable option. Welding affects the substrate, leading to a higher residual stress that may initiate secondary cracks. By contrast, laser cladding has a higher forming accuracy, a lower substrate thermal effect zone and better mechanical properties, apart from retaining the same quality and performance as the original condition. Laser cladding has also been widely used for repairing camshafts and turbine blades (Liu et al., 2016). The study focused on the environmental effect of laser cladding but provided no detailed explanation on the repair.

Buican, Oancea & Manolescu (2014) studied the applications of SLM technology in repairing the broken teeth of a sewing machine gear during remanufacturing. SLM enables direct melting of metal powders such as titanium, steel, chrome, cobalt and aluminium alloys, which are built using the layer-on-layer approach. In this method, the laser beam acts as the energy supplier to melt a layer of deposited metal powder and fuse it into the previously melted layer. With the use of AM, processes such as scanning, repairing and remanufacturing can be implemented within a day with the aid of technical information on the specifications of the damaged parts. The authors provided a detailed explanation on the reparation of gear teeth using SLM.

Yilmaz, Gindy & Gao (2009) studied and improved the remanufacturing of aero engine components using AM. Aero engine components such as blades, vanes, combustors and shafts are easily damaged due to exposure to harsh environments and long operating hours. Typical forms of damage to these components are distortions, wears, impacts dents and cracks. Damaged blades are restored to their original shape and efficiency because they are critical to the operations and performance of the system. Yilmaz, Gindy & Gao (2009) proposed reparation using laser cladding, followed by removal of the excess material using a conventional machine.

Researchers such as Al Handawi et al. (2020) highlighted the need to address limitations in AM repair from the perspective of environment, economics and social. Morrow et al. (2007) compared the economic and environmental effects of AM repair and conventional machining (CNC milling) in the tooling industry. Whilst both processes showed a trade-off between economic and environmental effects, AM repair fared better in terms of energy reduction and material inventory because AM requires only a specified amount of the material. Aziz et al. (2021) discussed the pros and cons of the welding method (conventional) and AM. Despite the limitations in both methods, the authors highlighted the contributions of AM to environmental sustainability such as environmental footprints from component manufacturing process can be reduced (Huang et al., 2016), the capability to extend the product’s life is enhanced (Den Boer, Lambrechts & Krikke, 2020; Ford & Despeisse, 2016) and the process is environmentally friendly (Aziz et al., 2021).

Liu et al. (2016) focused on the environmental effects from the remanufacturing of cast iron cylinder head block through laser cladding using life cycle assessment. In the study, the energy consumption for manufacturing and remanufacturing was collected. It was also reported that remanufacturing can reduce environmental impact by 63.8% on average. The steps involved in remanufacturing the cylinder head was discussed briefly and to conduct a scenario analysis, the crack on a worn cylinder head was assumed repaired. Based on the study, the energy consumption for manufacturing a single part of cylinder head is higher, 112.75 KWh, compared with the energy consumption for remanufacturing that is approximately 91.75 KWh. According to Faludi et al. (2016), 85% of energy is used for CNC machining including excess material removal after processing. However, AM can save energy of up to 45%–61% based on type of process and machine used, which is 45% for Thermojet, 43% for EOS and 61% for FDM.

Despite the growing interest in automating repair using AM, reports on the requirements from product, process and system perspectives are limited. To date, the practical applications of AM for repair are still limited, especially in developing countries due to the high acquisition and maintenance cost of AM technology such as DED and the competencies needed to handle such machines.

### Application of intelligent systems in manufacturing

Recent literatures for example by Rezaei, Houshmand & Fatahi Valilai (2021) highlighted the application of AM, AI and cloud manufacturing as pillars for digitised IR. The authors used convolutional neural network to interpret geometrical shapes of 3D objects from 2D pictures for categorisation into simpler shapes and analysed their production automatically using AM. The proposed method can reduce cost and time in addition to creating a fully automated platform in the manufacturing system.

Sadeghi Aghili et al. (2021) proposed the application of cloud manufacturing and service-oriented process to enable the transition of conventional manufacturing business model into a more efficient and productive one by using algorithm and solve the scheduling problems of logistics and manufacturing. Industrial AI for Industry 4.0 in manufacturing was proposed by Lee et al. (2018) as a solution for real-time monitoring and performance prediction of machine tool spindle in CNC machine. The proposed AI application is capable of minimising maintenance cost and optimising product quality simultaneously. For the purpose of this paper, the application of AI technology is focused on repair and restoration during remanufacturing.

### Application of intelligent systems in AM repair and restoration

The current production systems are more complex, dynamic and chaotic in behaviour, but fulfilling customer demand through high-quality production based on ML in intelligent production systems is necessary. According to Wuest et al. (2016), AI is one of the most interesting developments of ML. Kashyap (2017) highlighted the widespread applications of AI in demand forecasting, error detection, equipment failure prediction and pattern recognition. AI is also important for predicting machinery failures and maintenance, optimising energy consumption, predicting part failure and effective decision making (Kashyap, 2017).

AI provides various algorithms, theories and methods, and offers great potential to transform the current manufacturing technique under the situation of ever increasing data repository. Through ML in manufacturing, useful information can be derived from existing data sets, thus providing a basis for approximations or predictions that allow machines to operate with future behaviours such as decision making and automatic system improvement. It is also beneficial for detecting certain patterns or exploring regularities in a dynamic manufacturing environment (Wuest et al., 2016).

ML can be defined as the process of computers being taught the ability to extract important information from examples automatically or the capability of machines to build on the strength of big data to optimise the process, find new solutions and gain new insights. It can make predictions based on a large amount of data and process the ability to recognise patterns and learn from experience (Ma & Okudan, 2015). Ansari, Erol & Sihn (2018) noted that ML can improve quality through systems training using large datasets, whereas humans can be influenced by personal, societal or institutional interests during decision making. Moreover, humans become fatigued and dissatisfied with their job, whereas machines can operate for countless hours. Table 4 presents a summary of selected articles on the application of AI techniques in damage and failure detection.

**Table 4:** Applications of AI-based techniques in repair and restoration. Summary of the work reported in the related articles

No	Author	Topic	Year published	Description of the published works
1	Xiang et al.	Crack Detection in a Shaft by Combination of Wavelet-Based Elements and Genetic Algorithm	2008	• Detection of crack location and depth in shafts using GA kand B-spline wavelet-based elements. • Rotating Rayleigh-Euler and Rayleigh-Timoshenko beam elements of B-spline wavelet were constructed on the interval to discretise slender shaft and stiffness disc in order to gain accurate frequency. • The experiment was modelled using wavelet-based elements to gain precise frequency. The measured frequency was used to detect crack while GA normalised the crack location and detected the depth of crack. • Numerical and experimental cases of cracked shaft were also conducted. • A comparison of predicted and actual crack parameters showed relatives errors of not more than 0.2%. • GA was applied to eliminate errors of frequency between numerical simulation and experimental measurement which indicated the success of the proposed method for detecting cracks in shaft with high performance.
2	Yang et al.	Gear Fault Diagnosis Based on Support Vector Machine Optimized by Artificial Bee Colony Algorithm	2015	• The use of Support Vector Machine (SVM) and artificial bee colony (ABC) algorithm for parameter optimisation in fault diagnosis of gearbox. • Experiments on gear vibration were performed on machinery fault simulator which involved several procedures such as data acquisition, feature extraction using ensemble empirical mode decomposition (EEMD) method and an improved method based on empirical mode decomposition (EMD). Parameters were then optimised using binary tree SVM mode and Gauss RBF kernel function. Results were verified and validated using GA and particle swarm optimisation (PSO). • The ABC algorithm was found to be suitable for SVM paramete optimisation of gearbox fault diagnosis, giving high accuracy and least time taken. • The recognition rates of gearbox faults were improved by using SVM classification model that was optimised based on ABC.
3	Yuwono et al. (2016)	Automatic Bearing Fault Diagnosis Using Particle Swarm Clusteringand Hidden Markov Model	2016	• Method for diagnosis of automatic bearing defects based on Swarm Rapid Centroid Estimation (SRCE) & Hidden Markov Model (HMM). • The detection involved several methods such as signal processing, features extraction and defect classification system. • The proposed method for defect detection achieved an average of 98.02% sensitivity and 96.03% accuracy in distinguishing the source of defects. Error rate was reported to be low at only 2.65%.
4	Chou, Ngo & Chong (2017)	The use of artificial intelligence combiners for modelling steel pitting risk and corrosion rate	2017	• Based on a theory of risk management, an AI-based model for prediction was used, which included single and ensemble models constructed from four well-known machine learners namely, artificial neural networks (ANNs), support vector regression/machines (SVR/SVMs), classification and regression tree (CART) and linear regression (LR). • Prediction accuracy was evaluated using two real-world prediction datasets of 62 samples as performed by Shi et al. (2011), by measuring the corrosion potential using alkaline solution and pitting potential using electrochemical. Besides, the datasets from Liu et al. (2005) were also used which is 46 samples of 3C steel in different seawater environment. Corrosion data was obtained using electrochemical technique. • Data measurement and analysis were performed using the proposed method. • The predictive performance was compared using root mean square error (RMSE), mean absolute error (MAE), mean absolute percentage error (MAPE) and synthesis index (SI). • The study suggested that a hybrid model is better in predicting the pitting corrosion risk that can be used to schedule maintenance process. This will reduce risks of structure failure and maintenance cost.
5	Nasiri, Khosravani & Weinberg	Fracture mechanics and mechanical fault detection by different methods of artificial intelligence: A review	2017	• Application of different approaches of AI to fracture mechanics and mechanical fault detection such as Bayesian Network, ANN, GA, FL and CBR. • Bayesian Network was successfully applied in fault diagnosis of vibration signals in time-domain for gear train systems and mechanical fault detection in vehicle. • ANN was able to predict crack growth direction on sheet material under the influence of second or more cracks. Besides, ANN was also applied in fault diagnosis of rotating parts and machinery, and in fault and error detection on helicopter rotor system. • GA was used to locate and determine the depth of cracks in shafts besides detecting multiple cracks on beam-like structures. • FL was used to predict and estimate failure in oil and gas pipeline, classify different faults on roll bearing and diagnose faults of motor bearing. • CBR technique was used to diagnose faults in the acoustic signals of industrial robots. Besides, CBR was also applied in fault diagnosis of injection moulding machine as well as gas and turbine steams during maintenance. The CBR technique was able to reduce maintenance cost and help users in decision making during diagnosis and maintenance of gas turbine.
6	French, Benakis & Marin-Reyes	Transfer Analysis of Human Engineering Skills for Adaptive Robotic Additive Manufacturing in the Aerospace Repair and Overhaul Industry	2018	• Repair and overhaul in the aerospace industry using AM by demonstrating the design process and analysing the output source from observations on highly skilled human engineer during manual remanufacturing and repair technique. • Comparison between manual process monitoring and real-time weld monitoring for process parameters. • During the welding process, variations in power supply will result in the reduction of heat input to material, causing lack of fusion in weld. Using a real-time weld monitoring system, the slight variation of the power supply will be detected which triggers a signal for a change in the process parameters. Another subsystem will adjust the power to its new level. By doing so, the process continues with the corrected parameters and prevent defects due to lack of fusion. • The design principles enabled the robotic system to adapt to changes in the repair process, resulting in better quality and high success rate. • The authors developed an automatic voltage control (AVC) module to achieve machine control of the arc welding voltage.
7	Francis and Bian	Deep Learning for Distortion Prediction in Laser-Based Additive Manufacturing Using Big Data	2019	• The application of Deep Learning approach in predicting distortion for geometrical accuracy of fabricated parts in Laser Based Additive Manufacturing (LBAM). • A disk was fabricated using Optomec LENS™ 750 systems using Ti-6Al-4V powder and its distortion was measured using Talysurf CLI 3D surface profiling system. • The proposed methodology was able to increase the accuracy of distortion prediction leading to an increase in the overall geometric accuracy of LBAM.

According to Liu et al. (2018) the main goal of AI is for human and machine to be integrated and coexist harmoniously to ensure efficiency in carrying out tasks through the implementation of intelligent machine. As for Industry 4.0, which is highly related to smart factories, it will be controlled physically by machine intelligence for autonomous, flexible manufacturing purposes embedded in the planning, production and management processes for real-time decisions (Terziyan, Gryshko & Golovianko, 2018). The implementation of big data technology will create a revolution of traditional technology that will improve product quality, increase production efficiency and reduce energy consumption (Chen et al., 2018).

French, Benakis & Marin-Reyes (2018) discussed the implementation of robotic AM in aerospace maintenance, repair and overhaul of components, such as vanes, turbine blades and compressor blades, aimed at retaining the functionality and long-term reliability of the components. The authors proposed that AM, which is a series of welding trials consisting of monitoring welding, predicting quality and detecting the occurrence of errors and damage, be automated. In addition, a robotic welding system, which will soon be able to reshape the aerospace remanufacturing industry, can be implemented based on Industry 4.0 principles. According to the concepts of Industry 4.0, robotic systems that can adapt to changes in the repair process can provide better quality control and a higher success rate. A smart factory can also increase production speed and have an excellent quality assurance. The study detailed repair and overhaul activity in the aerospace industry. The authors also noted that developing this high technology for future automotive component repair and restoration is possible. The approach will reduce time, waste, production costs and dependency on a highly skilled workforce for selected repair processes.

To date, AI has been widely deployed in various applications of component repair. Table 4 provides a summary of the work reported in the related articles.

### Applications of AI-based techniques in repair and restoration

Kashyap (2017) highlighted the widespread applications of AI in error detection, equipment failure prediction and pattern recognition. Nasiri, Khosravani & Weinberg (2017) noted that artificial neural networks (ANNs) have been used to detect faults in the rotor system of helicopters and can predict crack growth for sheet material. Fang et al. (2020) reported on the use of deep learning in malware detection that achieves better results with a high effectiveness. Moreover, feature extraction for malware detection performs better with deep learning (Fang et al., 2020).

The artificial bee colony (ABC) algorithm was applied by Kurilova-Palisaitiene, Sundin & Poksinska (2018) in the detection of faults in the gearboxes of mechanical equipment. The conventional methods used in gearbox fault detection are time domain, frequency domain and time–frequency domain. The authors proposed an integrated system based on support vector machine and ABC, and their study showed that the proposed method took less time to collect vibration signals for the diagnosis of gearbox failure than the conventional method. AI techniques have also been used in the fault diagnosis of automatic bearing using a combination of particle swarm clustering and the Hidden Markov Model. The application proposed by Yuwono et al. (2016) involved a mathematical model for the proposed framework, and the experiment showed that the hybrid techniques distinguished the defects with a lower error rate.

CBR is an AI technology for problem solving that relies on learning and reasoning based on previous experience (Yang et al., 2015). This technique has also been used for detecting mechanical failures in a component (Yuwono et al., 2016). The approach can reduce dependency on extensive knowledge and information on failure analysis. According to Nasiri, Khosravani & Weinberg (2017), the CBR technique has been used for the detection of leakage in gas and steam turbines, which helps in decision making, whilst reducing maintenance costs and increasing the efficiency of expert systems. Xiang et al. (2008) successfully applied GA in the detection of crack locations on shafts and the depths of the shafts. All these techniques can be integrated with ML for detecting component failure and its severity, such as corrosive surfaces, cracks, dents and scratches during repair and restoration in remanufacturing.

Francis & Bian (2019) proposed an intelligent system based on deep learning for laser-based additive manufacturing (LBAM). The aim of the system is to overcome challenges in geometric accuracy to predict distortion accurately. Based on the system, distortion that usually occurs during part fabrication was predicted on various geometries for end-use applications. The accuracy and efficiency of LBAM was improved after using this approach. The output of the study was also an enabler for Industry 4.0. According to the authors, the method cannot be directly applied on the boundary of complex parts. For complex parts, a combination of more than one method, such as combining the proposed method with ANN was proposed. Kurilova-Palisaitiene, Sundin & Poksinska (2018) stated that lack of automation in companies and the complex geometry of components are amongst the challenges that cause inefficiencies in remanufacturing.

French, Benakis & Marin-Reyes (2018) proposed a system that used a robotic arm system to automate the repair of an aerospace component, which is the tip of a blade that has complex geometries and different repair requirements. This tip repair relied heavily on highly skilled welding engineers who use GTAW-based AM in repairing. The proposed system is an intelligent 3D scanning that will ensure that the surface quality meets the requirements for repair to calculate in parallel the AM deposition path of the system. The working principle of the system is the transparency of information between suppliers, manufacturers and customers, which has a recording of its history since manufacturing. According to the authors, the system can provide detailed reports on each individual manufactured component. The online process monitoring, the concept of industrial informatics and the component regeneration process will then be transformed into cyber–physical systems to obtain an optimum weld quality.

## Discussion and Future Research Directions

Through remanufacturing which is one of the EoL recovery strategies of CE, EoL components are repaired and reused in their new life cycle in an as-good-as-new condition. To enable efficient repair using AM, the design of components has to be planned to support such recovery strategies and methods from the perspectives of design features and geometrical complexity, material compatibility and process system capabilities. 3D printing techniques, or AM technology will make design options of products become countless because the technology allows the creation of complex geometries whilst reducing material usage and energy consumption (Huang et al., 2014). Recent advancements in AM have further leveraged its potential in component repair and restoration. Through AM integration with AI technology in ML, human thinking and decision making can be replicated to enhance design and process efficiencies in repair and restoration, thus improving the circulatory of materials and products. CE is a potential solution in minimizing the exploitation of resources and maximizing the prevention of waste to make better use of resources (Velenturf & Purnell, 2021).

According to Macarthur (2019), technology such as AI can unlock CE opportunities by improving design to empower cycles of reuse, repair, refurbishing and recycling of technical materials. According to Akinode & Oloruntoba (2020) AI is a key driver of IR 4.0 that can bring dreams of CE vision to fruition. Ghoreishi & Happonen (2020) stated that AI enables the transfer of precise data and information on materials and product availability, condition and accessibility that will lead to ease of monitoring. This process will lead to remote maintenance as well as remanufacturing, reuse and repair opportunities.

As reported in the earlier section, AI has been deployed in various applications of repair and restoration from detection of errors, defects and failures in components and machines to the diagnosis and prediction of failures in parts and components. AI techniques deployed in the earlier work include genetic algorithms, artificial neural network, fuzzy logic, case-based reasoning, Bayesian network and their hybrid techniques. Most of the studies have focused on determining the accuracy and precision of the developed techniques. The knowledge and heuristics involved in the process of decision making were not discussed at length. Knowledge and heuristics are crucial aspects of human judgement and decision-making during the repair and restoration, more so in remanufacturing when products are expected to be returned to their original specifications.

During a conventional process of repair and restoration, workers have to assess the conditions of the cores that include types of damage, severity of damage, geometric complexity and availability of the right tools. Each incoming core has different levels of conditions and geometrical complexity, causing vagueness and uncertainties in decision making. AI techniques such as fuzzy logic, ANN, CBR and their hybrid methods can represent this human decision making. The output from such systems will be useful for component design optimisation to facilitate repair using AM. A fuzzy-based system for example, comprising rules on the conditions of the EoL components should be able to assist in deciding whether components can be repaired and returned to their original specification. The basic system will require appropriate sensors for component and failure detection to be integrated with such fuzzy-based systems. Optimisation methods such as genetic algorithm and other evolutionary optimisation methods can be further deployed for process and cost optimisation.

AI-based optimisation methods have grown exponentially over the last decade. The integration of AI techniques in repair and restoration using AM should be able to support optimisations in product design to facilitate repair using AM. Meanwhile, process optimisation is necessary to ensure repair effectiveness and cost efficiency through the use of predictive techniques such as neural network, hence remanufacturing worthiness and economic returns of the repaired component can be ascertained.

The following discussion as itemised below, provides insights from the literature review whilst highlighting remarkable issues and opportunities for further enhancement of automated repair and restoration using AM through the integration of AI systems.

1. Manual repair and restoration of components or parts in remanufacturing requires skilled and experienced workers to make effective decisions to ensure that remanufactured parts are returned to their original specification. Decisions are influenced by the conditions of the incoming cores such as mode of failures, severity and locations of damages, difficulty in reparation due to geometrical complexity and use of nonstandard components, and selection of the appropriate repair processes or tooling. Factors such as unavailability of skilled, experienced workers and human limitations such as inconsistency in the quality of output, are several of the reported challenges in remanufacturing. Automating the repair process with the support of intelligent systems should be the way forward, as it is crucial to ensure efficiency and consistency in the quality and the reliability of the remanufactured components. Through AI, product design data and information can be captured and shared within a smarter remanufacturing environment.
2. Advancements in AM technology have extended the conventional role of AM from part fabrication to part repair and restoration. DED and laser cladding technology have been widely reported as the most capable AM technology for conducting repair and restoration. Whilst the technology is relatively new and the cost is high, several papers have reported the advantages of AM in repair efficiency. The contribution of AM to environmental sustainability has also been highlighted by several researchers.
3. Despite the potential and the benefits of AI in component repair, its practical applications in the remanufacturing industry have not been widely reported. The integration of AI in AM repair should be able to provide an intelligent platform for the remanufacturing industry, but its implementation will certainly require an in-depth study to counter upcoming challenges in identifying requirements, for example, the right setting of process parameters for various conditions of the incoming cores. Incoming cores have different modes of failure, severity of damages and occurrences of damage at different locations of parts or components. Verifying the quality of reparation is also crucial for the system because remanufactured components have to be returned to a ‘like-new’ condition. Through algorithms and enablers such as sensors, failures or damages of incoming cores can be diagnosed, and the quality of repaired components can be ascertained effectively.
4. To date, AI has found widespread applications in the diagnosis, prediction and detection of failures or damages in parts and components. Knowledge-based techniques such as fuzzy inferencing and CBR when hybridised with learning algorithms such as neural network and deep learning will certainly provide an intelligent system based on ML. The system should be able to assist in design optimisation for different component failures and damages to be repaired using AM. Genetic algorithms and other more recent optimisation techniques such as particle swarm optimisation and ant colony optimisation can be deployed to optimise design parameters for efficient, effective repair. Moreover, a hybrid system should be able to offer a better replication of human thinking and decision making for design optimisation through ML.
5. The development of an intelligent system for component repair will require human knowledge and experience related to component failures, design and geometry, materials and repair processes. These knowledge and heuristics must be acquired from the so-called experts to ensure that the system will successfully automate the repair process not only in terms of effectiveness but also cost efficiency. Whilst the acquisition cost of AM technology capable for repair is still high, cost–benefit analysis must be carefully considered to ensure return on investment.
6. Whilst recent literature has reported on process system capabilities of advanced AM technologies for component repair, limited reports have focused on the design aspects of high-value remanufacturable components that can be repaired using the AM technologies. AM technologies are highly capable of building complex shapes and lightweight structures, but ease of repair on components with complex shapes using AM has not been thoroughly addressed by researchers. Guidelines and principles on design and optimisation of components for AM manufacturing have grown exponentially over the past few years, but guidelines and principles on design and optimisation of components for AM repair are still at the nascent stage. This aspect of designing products for life cycle extension is crucial to assist the designers in ensuring their products will be repaired and restored effectively and efficiently through automated repair using AM.

## Conclusion

Remanufacturing is one of the EoL recovery strategies of CE that can sustain natural resources by reducing raw materials and energy consumption compared with the production of a new product. Remanufacturing is responsible for extending the life cycle of products by returning them to their original specifications and warranty. The review shows that automation of repair using AM can improve efficiency and overcome issues related to conventional repair, such as dependency on skilled workers and issues related to inconsistency in the output quality. Since EoL component repair for remanufacturing is highly dependent on human judgement and decision-making, it is necessary for the automated system to be integrated with AI capabilities. Knowledge-oriented techniques of AI can be used in the acquisition and optimisation of knowledge and heuristics from highly skilled and experienced workers. Such knowledge does not only support process optimisation in repair and restoration but also optimisation and improvement to product design in order to support the efficiency and effectiveness of the repair process. Thus, AI play a remarkable role in supporting automated repair and restoration achieve the principles of CE to extend the life cycle of products. The outcomes of this review are also expected to broaden research in the field of Design for Additive Manufacturing Repair, which has a direct contribution to the implementation of CE.

## Additional Information and Declarations

### Data Availability

The following information was supplied regarding data availability: There are no raw data involved in this study.
